# Pachychoroid Spectrum Disorder Findings in Patients with Coronavirus Disease 2019

**DOI:** 10.1155/2021/4688764

**Published:** 2021-09-13

**Authors:** Mojtaba Abrishami, Ramin Daneshvar, Nasser Shoeibi, Neda Saeedian, Hamid Reza Heidarzadeh, Seyedeh Maryam Hosseini

**Affiliations:** ^1^Eye Research Center, Mashhad University of Medical Sciences, Mashhad, Iran; ^2^Department of Internal Medicine, Faculty of Medicine, Mashhad University of Medical Sciences, Mashhad, Iran

## Abstract

**Purpose:**

To report the occurrence of acute, bilateral, central serous chorioretinopathy (CSC), and pachychoroid spectrum disorder findings in patients with coronavirus disease 2019 (COVID-19).

**Methods:**

In recovered cases of COVID-19 with visual disturbances, complete ocular examinations with multimodal retinal and choroidal evaluation, including enhanced depth imaging optical coherence tomography, fluorescein or indocyanine green angiography, and blue autofluorescence, were obtained.

**Results:**

Four COVID-19 recovered patients presented with bilateral blurred vision. Ocular examination and imaging revealed pachychoroid and pachyvessels associated with choroidal hyperpermeability without any obvious intraocular inflammation. Bilateral localized serous retinal detachment was obvious in three cases compatible with pachychoroid associated with CSC manifestation and pachychoroid pigment epitheliopathy in one patient. CSC was resolved with treatment by steroidal antimineralocorticoid (Eplerenone) in two patients and by photodynamic therapy in one patient. None of the patients reported emotional stress and history of corticosteroid consumption.

**Conclusion:**

Hyperpermeability of the choroid, pachychoroidopathy, or choroidal vessel congestion can be observed or exacerbated in association with COVID-19.

## 1. Introduction

Coronavirus disease 2019 (COVID-19) affects different parts of the body. The ocular findings in this regard were primarily confined to conjunctivitis [1, 2]. However, recent reports have indicated that the retina could also be involved in COVID-19 with the acute vascular lesions of the inner retina, including flame-shaped hemorrhages and cotton wool spots [2, 3]. Further peripapillary retinal vascular involvement in early post-COVID-19 patients has been recently described [2]. Moreover, viral ribonucleic acid (RNA) of severe acute respiratory syndrome coronavirus-2 (SARS-CoV-2) was found in the retinal autopsy sample of deceased cases of COVID-19 [4]. Presence of angiotensin-converting enzyme type 2 (ACE2), as the main receptor for SARS-CoV-2 in the retina, and the presence of its homology, ACE, in the choroid and different cell types of the retina [5], increase the possibility of chorioretinal involvement in patients with COVID-19.

Pachychoroid spectrum encompasses a group of different diseases which consist of central serous chorioretinopathy (CSC), pachychoroid pigment epitheliopathy (PPE), polypoidal choroidal vasculopathy (PCV), pachychoroid neovasculopathy (PNV), peripapillary pachychoroid syndrome (PPS), and focal choroidal excavation (FCE) [6]. CSC is a type of serous retinal detachment that mainly occurs in young to middle-aged males. CSC is usually accompanied by leakage at the level of the retinal pigment epithelium, which can be observed on fluorescein angiography (FA) and indocyanine green angiography (ICGA). Choroidal vascular congestion, prominent venous dilation, and hypercyanescent patches indicating areas of choroidal hyperpermeability are other features in angiographies in pachychoroid spectrum findings [6]. Enhanced depth imaging (EDI) optical coherence tomography (OCT) demonstrates that the choroid is pathologically thickened, and vascular lumens have significantly larger hyporeflective spaces. Generally, it is believed that choroidal hyperpermeability and choroidal vascular changes are the primary pathogenic steps and result in choroidal thickening [6]. There is evidence of the effect of the renin-angiotensin-aldosterone system (RAAS) on pachychoroid disease spectrum diseases [7–9], and as SARS-CoV-2 primarily affects the RAAS, there might be a relationship between these diseases. In this regard, herein we report acute bilateral CSC and pachychoroidopathy-like disorder in confirmed cases of COVID-19.

## 2. Case Presentation

### 2.1. Case 1

A 21-year-old male patient with a history of hospital admission for COVID-19 experienced a decrease in vision and metamorphopsia. He had a history of fever, cough, dyspnea, myalgia, red eye, and eye discomfort for two weeks. Results of his lab tests indicated lymphopenia, increased erythrocyte sedimentation rate (ESR) (48 mm/first hour), and increased C-reactive protein (CRP) (192.3 mg/L). Moreover, the real-time reverse transcription polymerase chain reaction (rRT-PCR) amplification of SARS-CoV-2 virus RNA from a nasopharyngeal (NP) swab sample was positive. He was admitted to the hospital for further supportive treatment due to tachypnea (22 breaths per min), decreased blood oxygen saturation (91%) on room air, and diffuse bilateral ground glass opacities in chest high-resolution computed tomography (HRCT). However, there was no need for intensive care, and after four days, the patient decided to continue his treatment in self-isolation at home. He was further treated at home with oral enteric-coated Naproxen and Azithromycin for one week and did not use any kind of systemic corticosteroids. On the eighth day after discharge, he experienced blurring of vision and metamorphopsia; however, there was no further red eye and ocular discomfort. In his ocular examination, the best corrected visual acuity (BCVA) was 20/25 in the right eye and 20/40 in the left eye.

**Figure 1:**
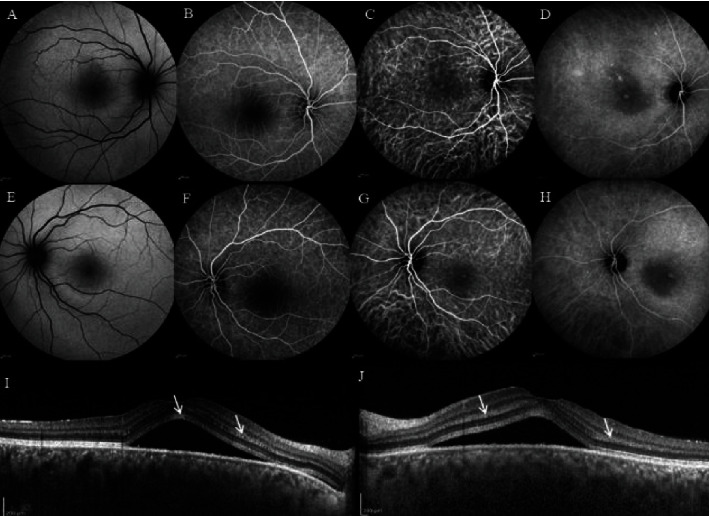
(a, e) Fundus autofluorescence (FAF), (b, f) fluorescein angiography (FA), (c, d, g, h) indocyanine green angiography (ICGA), and (i, j) enhanced depth imaging optical coherence tomography (EDI-OCT) of the first case. Retinal hyperreflective bands in different layers of the retina, serous retinal detachment (SRD) at the macula, and increased choroidal thickness on EDI-OCT can be observed. FAF and FA were nearly normal, and the ICGA showed a single hot spot at the center of the fovea. This spot appeared from the early phase and lasted until the late phase with multiple late hypercyanescence dots scattered at the macular area and outside of arcades, especially at superotemporal, and hyperpermeability of large choroidal vessels.

Furthermore, the intraocular pressure (IOP) and anterior segment slit-lamp examination were normal. In fundus examination, bilateral submacular fluid was obvious without any sign of intraocular inflammation. The EDI-OCT (Spectralis OCT manufactured in Heidelberg Engineering, Heidelberg, Germany) revealed retinal hyperreflective bands in different layers of the retina, serous retinal detachment at the macula, and increased choroidal thickness (Figure 1).

**Figure 2:**
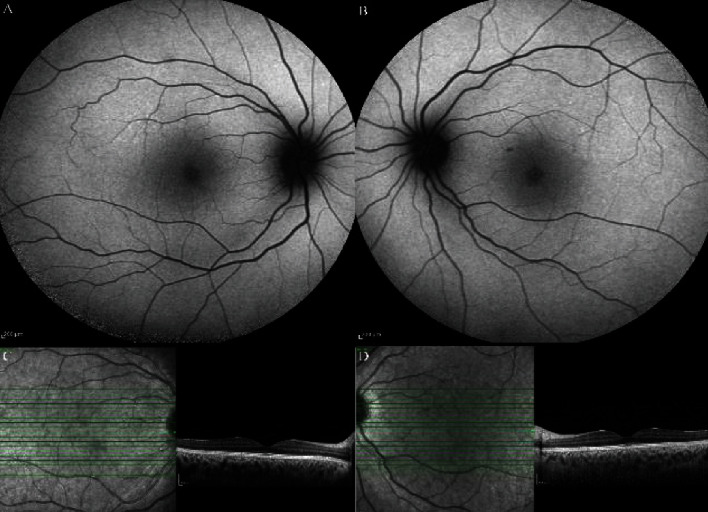
(a, b) Fundus autofluorescence and (c, d) enhanced depth imaging optical coherence tomography of the first case following three months of treatment with Eplerenone showing completely resolved SRD. The choroid seems to be still thickened.

Besides, the FA showed no significant leakage, specific signs of vasculitis, or ocular inflammation. On ICGA, in both eyes, a single hot spot at the center of the fovea appeared since the early phase and lasted until the late phase with multiple late hypercyanesence dots scattered at the macular area and outside of the arcades especially at the superotemporal quadrant of the fundus. Moreover, the hyperpermeability of large choroidal vessels was remarkable. After 3 months, CSC responded very well to Eplerenone 25 mg every 12 hours a day and SRF was resolved completely (Figure 2).

### 2.2. Case 2

A 34-year-old female patient with a history of outpatient treatment for COVID-19 referred with a complaint of paracentral scotoma in her left eye. She had a history of myalgia, chest pain, fever, red eye, and ocular discomfort for 10 days, and rRT-PCR for SARS-CoV-2 virus on a NP swab sample was positive. Based on laboratory evaluation, there were lymphopenia, increased ESR (32 mm/first h), and increased CRP (18 mg/L). The patient had been treated with Azithromycin and Acetaminophen at home in quarantine for two weeks. She had not used systemic corticosteroids in the last two years.

**Figure 3:**
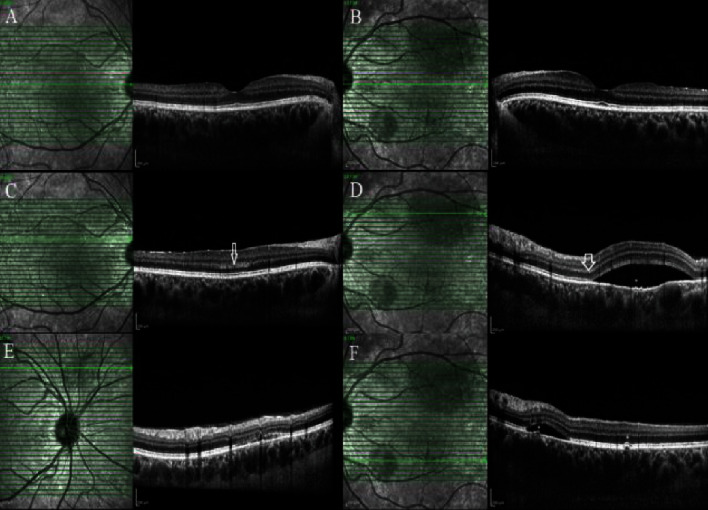
Enhanced Depth Imaging Optical Coherence Tomography of the (a, c, e) right and the (b, d, f) left eyes of the second case. Hyperreflective bands of the (c, d, arrows) outer plexiform layer, pachychoroid with dilated choroidal vessels and pachyvessels, and (d, f, asterisks) retinal pigment epithelium changes like pachychoroid pigment epitheliopathy can be seen in both eyes.

The patient experienced blurred vision in her left eye 10 days after the initiation of systemic symptoms. Visual acuity was 20/20 in both eyes, and according to the slit-lamp examination, the anterior segment was normal. In funduscopic evaluation, two small foci of serous retinal detachments in the macula and sparing of the fovea were found in both eyes. The EDI-OCT results revealed pachychoroid with dilated choroidal vessels in both eyes. Moreover, there was retinal pigment epithelial changes resembling pachychoroid pigment epitheliopathy and hyperreflective bands in the outer plexiform layer (Figure 3).

**Figure 4:**
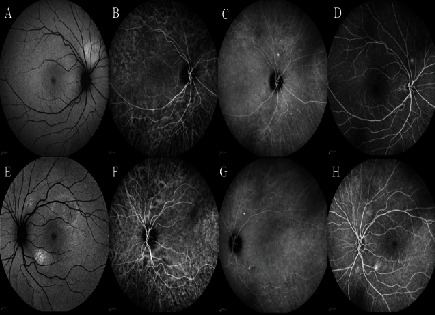
(a, e) Fundus autofluorescence, (b, c, f, g) indocyanine green angiography (ICGA), and (d, h) fluorescein angiography (FA) of the (a–d) right and the (e–h) left eye of the second case. The fundus autofluorescence demonstrated hyperautofluorescence foci corresponding to the leakage areas. The FA disclosed hyperfluorescent dots and leakage in both eyes. In the right eye, a hyperfluorescent focus superior to the disc was found on FA and fundus autofluorescence. The ICGA revealed dilation and hyperpermeability of choroidal vessels in the mid- and late phases, multiple small hypercyanescence dots out of the macular area in both eyes, and a hot spot corresponding to an inferotemporal SRD in the left eye.

Furthermore, two foci of subretinal fluid in the superotemporal and inferonasal area of the left fovea were found. In addition, FA results showed hyperfluorescent dots and leakage in both eyes as well as a hyperfluorescent focus superior to the disc in the right eye which also had increased autofluorescence. Further evaluations and EDI-OCT results confirmed the presence of a shallow serous retinal detachment with outer retinal changes. Bilaterally, ICGA demonstrated dilated choroidal vessels in the early phase followed by hyperpermeability of choroidal vessels in the midphase and multiple small hot spots out of the macula in the late phase. Moreover, there was a hot spot in the left eye which corresponded to the inferotemporal serous retinal detachment (Figure 4).

**Figure 5:**
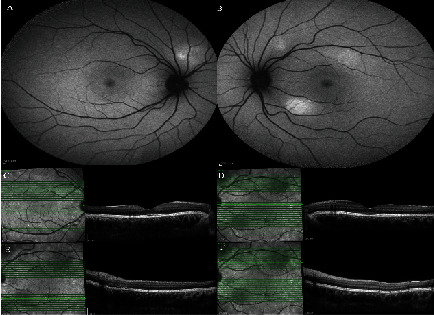
(a, b) Fundus autofluorescence and (c–f) enhanced depth imaging optical coherence tomography of the second case after two months of treatment disclosing completely resolved subretinal fluid in different parts of the macula, which were affected before. Choroidal thickening and dilation of choroidal vessels are still visible.

After 5 months, CSC responded to Eplerenone 25 mg every 12 hours and SRF was resolved (Figure 5). However, pachychoroid pigment epitheliopathy changes were not changed.

### 2.3. Case 3

A 41-year-old male patient with a history of outpatient treatment for COVID-19 came with a complaint of decreased vision in his right eye since one week after initiation of systemic symptoms. He had a history of severe head ache, myalgia, fever, and ocular discomfort for two weeks and positive rRT-PCR for SARS-CoV-2 virus on a NP swab sample. The patient became systemic symptom free one week after diminution in vision. Based on laboratory evaluation, there were lymphopenia, increased ESR, and increased CRP. The patient had been treated with Azithromycin and Naproxen at home in quarantine for two weeks. She had not yet used systemic corticosteroids.

**Figure 6:**
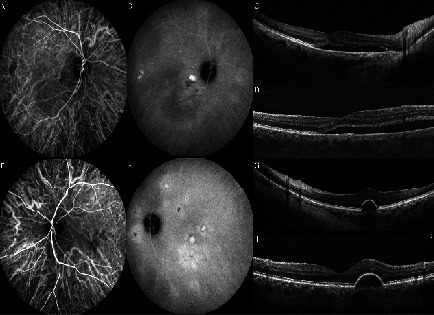
The indocyanine green angiography of the third case disclosed dilation and hyperpermeability of choroidal vessels in the early phase associated with multifocal hypercyanescence (hot spots) and diffuse leakage at the posterior pole in the late phase in the (a, b) right and the (e, f) left eyes. Enhanced depth imaging optical coherence tomography revealed (c, d) pachyvessels in the choroid and SRF in the macula of the right eye and (g, h) pigment epithelial detachment (PED) in the left eye.

Because of home self-quarantine, the patient came to us four weeks after decrease in his right eye vision. He also complained of gradual blurred vision and metamophopsia in his left eye. In examination, BCVA was 20/32 and 20/20 in the right and left eye, respectively. EDI-OCT revealed pachyvessels in the choroid and SRF in the macula of the right eye and pigment epithelial detachment (PED) in the left eye. ICGA showed bilateral dilatation and hyperpermeability of the choroidal vessels. Moreover, bilateral hypercyanescence as hot spots was obvious bilaterally (Figure 6).

**Figure 7:**
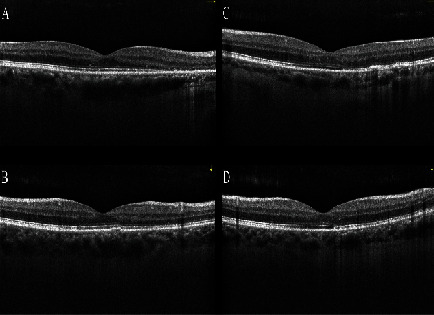
Optical coherence tomography of the (a, c) right eye and (b, d) left eye of the third patient one month after photodynamic therapy, even in the (a, b) horizontal or (c, d) vertical, shows complete fluid resorption and also outer retinal atrophy.

Photodynamic therapy (PDT) with half-dose of verteporfin was performed for both eyes. Two months later, SRF and PED were completely resolved with small defect in the outer retinal layers and BCVA was improved to 20/20 in the right eye (Figure 7).

### 2.4. Case 4

A 45-year-old female, a staff of eye hospital, with a history of outpatient treatment for COVID-19, was referred with a complaint of ambiguous visual disturbance in both eyes amid her disease period of systemic symptoms. She had a history of severe headache, myalgia, and fever for two weeks; rRT-PCR for SARS-CoV-2 virus on a NP swab sample was positive. Based on laboratory evaluation, there were lymphopenia, increased ESR, and increased CRP. The patient had been treated with oral Acetaminophen and Hydroxychloroquine at home in quarantine for ten days. She had not yet used systemic corticosteroids.

**Figure 8:**
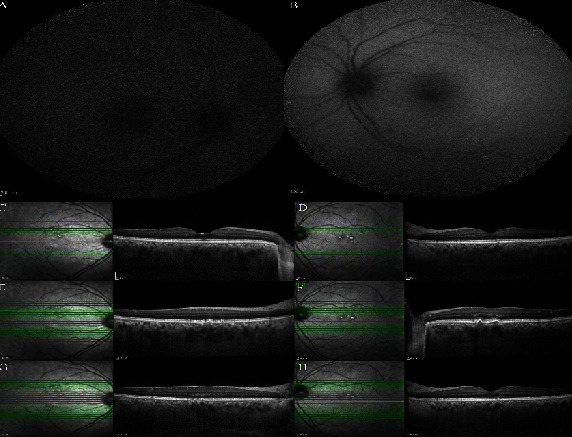
Fundus autofluorescence (FAF) and enhanced depth imaging optical coherence tomography of the fourth case. (a, c, e, g) The right eye images. (b, d, f, h) The left eye images. FAF of (a, b) both eyes disclosed no abnormality. In infrared reflectance taken with (c–h) OCT, bilateral hyperreflective dots were visible in both eyes. (c–h) Enhanced depth imaging optical coherence tomography depicted bilateral pachychoroid and pachyvessels accompanied by bilateral small, shallow, and multifocal retinal pigment epithelial (RPE) detachments and changes over the pachyvessels, hyperreflectivity of outer retinal layers, and multiple hyperreflective tiny dots scattered at the inner retinal layers in both eyes.

After return to her job, because of distorted vision, multimodal imaging was performed. Visual acuity was 20/20 in both eyes. She had not consent to take angiography. She had no history of abnormal vision or any visual abnormalities before COVID disease. In EDI-OCT, bilateral pachychoroid and pachyvessels accompanied by bilateral retinal pigment epithelial (RPE) changes over the pachyvessels and hyperreflectivity of outer retinal layers were obvious. Fundus autofluorescence (FAF) disclosed no abnormality. In infrared reflectance, bilateral hyperreflective dots were visible (Figure 8). The disease course was described for her, and she chose to be followed without any treatment.

## 3. Discussion

This study is a report on acute CSC and pachychoroid spectrum findings in confirmed cases of COVID-19. Despite the fact that conjunctivitis, chemosis, and retinal findings, including inner retinal changes, have been reported in COVID-19 cases [1, 3], however, to the best of our knowledge, there is no report regarding to the subretinal fluid, choroidal hyperpermeability, or choroidopathy similar to the pachychoroid spectrum diseases.

In the first patient with decreased vision, we found bilateral macular subretinal fluid (SRF) and retinal hyperreflective bands in OCT. Regarding to the almost normal FA results without any leakage, we proposed that the underlying cause of these presentations could be choroidal changes. On ICG, hypercyanescence target-shape spots at the fovea were in favor of leakage. Due to the lack of any corresponding leakage in FA, which is a typical finding in patients with CSC, we believe that the mechanisms of SRF a, in this case, might be a choroidopathy, perhaps in the context of viral involvement of the uvea similar to choroidal involvement in Flaviviridae viruses [10].

The second patient was a young female who presented with paracentral scotoma. Pachychoroid, pigment epitheliopathy, CSC, and pachyvessels were found to be associated with retinal fluorescein leakage foci that had correspondent hot spots in ICG angiography. Moreover, multiple tiny peripheral hypercyanescence spots were observed in both patients.

Given the history of COVID-19 in our cases, due to the presence of ACE and ACE2 in the retina and choroid [5], it can be postulated that the choroid may be involved either in the process of infection with SARS-CoV-2 or the cytokine storm in that setting. Multiple punctate hypercyanescent dots that were scattered at the macular area and beyond as well as the pachyvessels out of the macula were depicted on ICGA indicating a diffuse choroidal disease.

We did not find any sign of chronic CSC in the examination or autofluorescence results of our cases. Moreover, there were no significant pachyvessels on macular EDI-OCT despite the fact that the choroid had been thickened and the sclerochoroidal border was not visible beyond the imaging depth limit. Based on these pieces of evidence, it seems that choroidal hyperpermeability and increased thickness might be caused by an inflammatory process rather than only choroidal vessel dilations in the macular area or exacerbation of old pachychoroidopathy.

In the Flaviviridae family, a group of viruses consists of more than 90 RNA-enveloped viruses similar to the coronaviruses; choroidal involvement has been reported before [10]. It was hypothesized that the virus affects the vessels, and it can pass the blood-retinal barrier. As these viruses modulate retinal innate response, the protective barrier breaches and causes choroidal or retinal pathologies. Bilateral choroidal effusion has been reported from the Dengue virus, a member of this family [11]. Moreover, the multimodal imaging in Dengue fever maculopathy revealed hypocyanescence on the late frames of ICGA without fluorescein leakage accompanied with focal swelling of the outer plexiform and outer nuclear layers and discontinuity in the underlying ellipsoid zone in OCT [12].

Range of reported risk factors for the pathogenesis of CSC is large. Reported etiologies include pregnancy; endocrine disorders, such as Cushing syndrome; steroid-producing tumors; gastroesophageal disorders; psychologic stress; type A personality; organ transplantation; and multisystem autoimmune diseases, like lupus erythematosus. Medications that were found to be associated with CSC are corticosteroids, antihistamines, phosphodiesterase-5 inhibitors, MEK-inhibitors, sympathomimetic drugs, and antibiotics [6, 13]. In the cases of this study, nonsteroidal anti-inflammatory drugs (NSAIDs) were used since it has been reported that NSAIDs, whether topical [14, 15] or systemic [16, 17], could be effective in the treatment of CSC. Therefore, the use of NSAIDs for the cases of this study might have mitigated the occurrence of CSC. On the other hand, prostaglandin activity was observed in the RPE cells, as well as retinal vasculature [18]; hence, some physicians believe that NSAIDS can delay or prevent spontaneous recovery of CSC in a natural course [19]. However, there is not robust evidence regarding the detrimental effect of systemic NSAIDs on CSC. Therefore, the impact of NSAIDs on the genesis of CSC in these patients is improbable.

Moreover, none of the patients received corticosteroid therapy as these agents were not yet introduced into the COVID-19 treatment protocol at the time. Hence, the pathogenesis of CSC cannot be attributed to systemic corticosteroids. Furthermore, our patients received antibiotics that had been assigned as a risk factor for CSC in a retrospective case-control study [13]. It has been postulated that adaptation to stress, either as psychologic, neuroimmunological, neuroendocrine, or biochemical adaptations, is common in CSC patients. This could explain the higher rate of antibiotic consumption among CSC patients [13]. Furthermore, impaired immunity patients who experienced psychologic stress are more susceptible to infections [20, 21]. Hence, the antibiotic consumption itself may not consider as risk factor, and the confounding effect of stresses may affect more pronouncedly.

There are several plausible theories for SRF accumulation in these cases, including (1) stress caused by the fear of COVID-19 disease, (2) choroidal congestion and hyperpermeability secondary to the viral infection and as a component of systemic, multiorgan involvement in the course of COVID-19 disease [2], and (3) the recurrence of previous CSC. Although neither of our patients was apparently stressed about the disease, however, the first theory could be a possible etiology. Besides, no sign of previous CSC was found in the imaging, examination, or medical history of these cases, and there was also no sufficient evidence to support the third theory. In a recent case series regarding >the spectrum of pulmonary pathology in a cohort of 40 decedents by postmortem autopsy of 40 rRT-PCRs of confirmed COVID-19 cases, vascular congestion and hemangiomatosis-like changes were found in half of them [22]. Moreover, in another postmortem analysis, endotheliitis was found in several organs as a direct consequence of viral involvement, with evidence of the presence of viral inclusion structures, accompanying the host inflammatory response [23]. COVID-19 is associated with widespread microangiopathy; thus, changes of blood flow in the retina and choroid are potential. In recent case-control studies, alterations of retinal microvasculature are found subsequent to COVID-19 infections compared to healthy control groups [24, 25]. Moreover, in a case-control study in patients stratified to mild, moderate, and severe COVID-19 disease activity and normal controls, patients with moderate and severe disease had decreased macular vascular density (VD) on OCT-angiography (OCT-A) as compared with control subjects or even those who were asymptomatic or paucisymptomatic [26]. These findings raise the possibility of subclinical vascular alterations in individuals who have apparently recovered from COVID-19 infection, and the findings were shown to be associated with the severity of the COVID-19. It is therefore important to monitor these retinal microvasculature changes to ascertain their longitudinal course [27]. Given the presence of ACE and ACE2 receptors in the choroid and retina and with regard to the known role of RAAS in CSC [7, 9], we believe that the second theory is the most probable one. On the other hand, other differential diagnoses including inflammatory uveitic etiologies like Vogt-Koyanagi-Harada disease, age-related macular degeneration, melanoma, or other choroidal masses have been ruled out as these young patients without any other comorbidity, with pachychoroid-like choroidal changes, responded very well to Eplerenone and PDT.

In conclusion, we report four cases of acute CSC in association with COVID-19. It should be noted that in all cases, CSC was simultaneously bilateral. This might have been a simple coincidence or a pathophysiologic correlation between the two entities. Either way, regarding the novelty of COVID-19 and its many unknown faces, every practitioner managing these patients should anticipate various clinical pictures and organ system involvement and treat the variable clinical presentations of COVID-19 as a rule and not an exception. Regarding to accumulative evidences on retinal and choroidal changes due to COVID-19 infection in this report and previous reports [24–28], we could propose the COVID-19 infection as a new etiology for pachychoroid spectrum disorder.

## Data Availability

The datasets used during the current report are available and can be obtained from the corresponding author by a reasonable request.

## Abbreviations

COVID-19: Coronavirus disease 2019
OCT: Optical coherence tomography
SARS-CoV-2: Severe acute respiratory syndrome coronavirus-2
ACE2: Angiotensin-converting enzyme type 2
ACE: Angiotensin-converting enzyme
CSC: Central serous chorioretinopathy
ICGA: Indocyanine green angiography
EDI-OCT: Enhanced depth imaging optical coherence tomography
rRT-PCR: Real-time reverse transcription polymerase chain reaction
SRF: Subretinal fluid
RAAS: Renin-angiotensin-aldosterone system
PPE: Pachychoroid pigment epitheliopathy
PCV: Polypoidal choroidal vasculopathy
PNV: Pachychoroid neovasculopathy
PPS: Peripapillary pachychoroid syndrome
FCE: Focal choroidal excavation
ESR: Erythrocyte sedimentation rate
CRP: C-reactive protein
NP: Nasopharyngeal
HRCT: High-resolution computed tomography
BCVA: Best corrected visual acuity
IOP: Intraocular pressure
PED: Pigment epithelial detachment
PDT: Photodynamic therapy.
